# Effects of Increasing Farmed Salmon Intake to the Recommended Fish-Intake Amounts on Lipid Profile in Young Women: An 8-Week Intervention Study

**DOI:** 10.3390/nu16234051

**Published:** 2024-11-26

**Authors:** Zofia Utri-Khodadady, Dominika Głąbska

**Affiliations:** Department of Dietetics, Institute of Human Nutrition Sciences, Warsaw University of Life Sciences (WULS-SGGW), 159C Nowoursynowska Street, 02-776 Warsaw, Poland; zofia_utri@sggw.edu.pl

**Keywords:** smoked salmon, Salmo Salar, finfish, dietary intervention, fish recommendations, omega-3, EPA, DHA, cholesterol level, atherogenic index, cardiovascular disease

## Abstract

Background/Objectives: Habitual dietary changes that could help reduce the potential consequences of excessive body mass, such as hyperlipidemia and increased cardiovascular disease risk, are needed. The aim of this study is to assess the impact of a farmed-salmon-based dietary intervention on lipid profile parameters in young women with excessive body mass. Methods: The 8-week intervention involved 38 pair-matched women aged 18–30 years with excessive body weight defined as BMI ≥ 25.0 kg/m^2^. Participants were randomly assigned to the intervention (200 g of smoked salmon weekly) or the control group. Lipid profile parameters (total cholesterol (TC), high-density lipoprotein (HDL), low-density lipoprotein (LDL), triglycerides (TAG)), as well as atherogenic indices (Atherogenic Index of Plasma (AIP), Atherogenic Coefficient (AC), Cholesterol Index (Cholindex), Castelli Risk Index 1 (CRI-1), Castelli Risk Index 2 (CRI-2) and the TAG/HDL ratio) were assessed before, during, and after the intervention. Results: At baseline, 47% of participants had at least one of the lipid profile parameters outside the recommended range. No statistically significant differences were observed in the TC, HDL, non-HDL, LDL, or TAG concentrations or in the various atherogenic indices between the intervention and the control group after the 8-week-long intervention. However, differences in the change of the LDL concentration were noted, with a decrease of 8.2 ± 20.7 mg/dL in the intervention group compared to an increase of 9.5 ± 20.0 mg/dL in the control group (*p* = 0.011), as well as in the change of the Cholindex for which a median decrease of 4.4 mg/dL was noted in the intervention group, while a median increase of 0.8 mg/dL in the control group was observed (*p* = 0.040). Additionally, across participants with a waist-to-height ratio (WHtR) greater than 0.5, an increase of 50.0 ± 68.3 mg/dL in the intervention group and a decrease of 8.6 ± 56.6 mg/dL in the control group was noted for the TAG concentration change (*p* = 0.040). Conclusions: Concerning the observed beneficial influence of increasing farmed salmon intake to the recommended fish-intake amounts on decreasing LDL and Cholindex in young women with excessive body weight after 8 weeks, it seems that such a diet alteration might be recommended for this group to decrease their risk of cardiovascular disease in the future. Nonetheless, regarding the diverse influence on TAG, further studies are needed to assess the influence of increasing the intake of fatty fish available on the market at present on human health.

## 1. Introduction

According to the statistics of the World Health Organization (WHO), excessive body mass is a global and still growing problem, as, based on data for 2022, globally, 2.5 billion adults are overweight and 890 million adults are obese; since 1990, the share of obese adults has more than doubled [1]. Excessive body mass leads to an increased prevalence of various health problems, with diabetes mellitus, cardiovascular diseases (CVD), hypertension, and hyperlipidemia being among the most significant consequences [2]. Recent meta-analyses have confirmed these risks across various populations, highlighting the increased likelihood of developing diabetes mellitus [3], CVD [4], and hypertension [5] in overweight and obese individuals, as well as positive changes in serum lipid levels following body mass reduction [6]. Moreover, a joint expert review of the Obesity Medicine Association (OMA) and the National Lipid Association (NLA), published in 2024 [7], emphasized that the indicated problems are linked with one another, as excessive body mass promotes hyperlipidemia, which is partly responsible for CVD development, while diabetes mellitus [8] and hypertension were previously indicated among other contributing factors [9].

It is important to note the higher prevalence of obesity in females compared to males, as well as the increasing incidence of excessive body mass with age, peaking between 50 and 65 years [10]. However, young adult women experience the highest rate of weight gain compared to other age groups, which is attributed to lifestyle changes, the transition to and through university education, and the use of contraceptives [11].

Given the associated health risks, there is a need for comprehensive dietary recommendations that could help reduce not only excessive body mass but also its potential consequences, such as hyperlipidemia, hypertension, and CVD incidence. The American Heart Association (AHA) explicitly includes fish and seafood in its guidelines, highlighting their role in combating some of these health risks. According to the AHA [12], consuming 2–3 servings of fish per week is associated with reduced mortality and a lower incidence of CVD, including coronary heart disease, myocardial infarction, stroke, and heart failure [13,14]. Additionally, the recent FAO/WHO background document on the risks and benefits of fish consumption indicates that there is evidence of an association between fish intake and reduced risk of CVD [15]. These benefits are primarily attributed to long-chain omega-3 fatty acids found in fish, particularly when fish replaces other animal-based products high in saturated fats [16].

However, a systematic analysis by Micha et al. [17], which included data from 266 countries gathered for over 20 years, revealed significant international variation in mean fish intake. This resulted in a wide range of seafood-derived omega-3 fatty acids consumption, that is, eicosapentaenoic acid (EPA) and docosahexaenoic acid (DHA) [18] consumption, from as low as 5 mg to as high as 3886 mg per day. Furthermore, the analysis indicated that only 45 countries had mean seafood-derived omega-3 fatty acid intakes that met the current guidelines of over 250 mg per day, a benchmark set by the European Food Safety Authority (EFSA) based on an analysis of national nutritional guidelines [19]. Notably, in 100 countries representing over 66% of the global adult population, the mean intake of seafood-derived omega-3 fatty acids was alarmingly low, at less than 100 mg per day. In Poland, the intake is only slightly higher, with an estimated average of 107 mg per day [17].

In recent years, various studies assessing the association between fish intake and CVD risk [20], as well as the impact of increasing fatty fish intake on lipid biomarkers [21], have been conducted. According to a pooled analysis of 4 international cohort studies of almost 200,000 people from 58 countries by Mohan et al. [20], fish intake of at least 175 g per week is associated with a lower risk of major CVD among patients with prior CVD, while according to a meta-analysis of 14 intervention trials of more than 1300 individuals by Alhassan et al. [21], consuming oily fish led to significant improvements in triglycerides and high-density lipoprotein levels. What should be noted, however, is that the mean age of the participants in the study by Mohan et al. [20] was 54.1 ± 8.0 years, and it concerned patients with prior CVD, while the meta-analysis by Alhassan et al. [21], despite including studies also conducted among young individuals and those with overweight and obesity, included studies published only until 2014. This is of importance, as it is indicated that in recent years, the diets given to farmed fish, such as salmon, have changed toward more plant-based, which is known to result in decreased sum of EPA and DHA (EPA + DHA) and increased omega-6 fatty acids [22], which might alter the effects of increasing the consumption of fish on human health at present. Therefore, new intervention studies with fish available on the market now are needed.

As described above, such new studies should particularly focus on young women [10], who experience the highest weight gain rate compared to other age groups [11] and are, therefore, vulnerable to the risks and consequences of CVD in the future. In light of this, the aim of the study was to assess the impact of a farmed-salmon-based dietary intervention on lipid profile parameters of young women with excessive body mass.

## 2. Materials and Methods

### 2.1. General Information

The study was carried out at the Dietetic Outpatient Clinic of the Department of Dietetics, Warsaw University of Life Sciences (WULS-SGGW), Poland. It followed the principles outlined in the Declaration of Helsinki and received approval from the Ethics Committee of the Faculty of Human Nutrition and Consumer Sciences of the Warsaw University of Life Sciences (No. 27/2018) and the Ethics Committee of the Central Clinical Hospital of the Ministry of the Interior and Administration in Warsaw (No. 2/2021). Written informed consent for participation in the study and all its procedures was collected from all study participants, and they were informed that they could withdraw at any time. The study procedures were conducted from November 2021 to February 2022, while the intervention lasted 8 weeks (from December 18 to February 11).

### 2.2. Studied Group

The recruitment for the study was conducted using the convenience sampling method. Invitations to participate were shared through university social media channels, and snowball sampling was also employed to enhance recruitment. The inclusion criteria were as follows: females aged 18–30, classified as overweight or obese (defined as Body Mass Index (BMI) ≥ 25.0 kg/m^2^ [23]), residing in Warsaw or its surroundings (to facilitate multiple visits to the Dietetic Outpatient Clinic at the Department of Dietetics, Warsaw University of Life Sciences (WULS-SGGW)), and providing written informed consent. Exclusion criteria included pregnancy, lactation, adherence to a vegan or any fish-excluding diet, allergies to fish or seafood, and using omega-3 supplements.

### 2.3. Dietary Intervention

The intervention involved the consumption of 200 g of smoked salmon weekly in order to increase the omega-3 fatty acids intake (especially the EPA and DHA). The smoked salmon was produced from farmed Atlantic salmon (*Salmo salar*), a species classified in a FAO/WHO expert consultation as ranking in the first and second groups for having the lowest concentrations of mercury and dioxins, respectively [24]. Additionally, it is high in EPA and DHA, which led the FAO/WHO to conclude that the benefits of consuming farmed Atlantic salmon outweigh the potential risks posed by these contaminants [24].

The consumption of 200 g of salmon per week aligns with fish intake recommendations, which in most European countries and the United States of America are approximately 150–300 g weekly [25]. This amount was also determined to be potentially easily incorporated into a habitual diet.

It was decided to use smoked, not fresh, salmon due to the longer shelf-life of smoked salmon, as well as the fact that smoked salmon can be consumed without any thermal processing; hence, its regular consumption is easier and quicker compared to the consumption of fresh salmon.

The smoked salmon used in the study came from a single producer, Suempol Polska Ltd., one of Poland’s leading salmon sellers. The salmon, sourced from Norway, was obtained from the same batch and packaged in identical sliced trays with modified atmosphere packaging. The ingredients of the smoked salmon used in the intervention were Atlantic salmon (*Salmo salar*) and salt.

Due to the large variations in the different fatty acids found in salmons [26,27], their content in the used product was measured by GBA Polska—a Polish food testing laboratory (accreditation number AB 1095 [28]; accredited since October 2009). The nutritional value of the smoked salmon used in the intervention is presented in Table 1. The average daily portion of smoked salmon based on 200 g per week (approximately 28.6 g) provided around 50 kcal, 5.8 g of protein, and 2.8 g of fat, including 403 mg of omega-3 fatty acids, out of which the EPA + DHA amounted to 174 mg, which corresponds to 69.6% of the daily 250 mg recommended by the EFSA [19].

The smoked salmon was given to the participants at no cost, as it was financed within the research, to enhance compliance with the intervention and ensure that the participants consumed fish with consistent nutritional value. The participants in the intervention group were given 200 g of smoked salmon per week, divided into four 50 g packages. Due to its long shelf-life, participants collected the products once every two weeks (receiving eight 50 g packages, i.e., 400 g in total) at the Dietetic Outpatient Clinic of the Department of Dietetics, Warsaw University of Life Sciences (WULS-SGGW), where the study was conducted. They were instructed to consume 4 packages (equivalent to 200 g) each week. They were allowed to consume the salmon in four separate 50 g portions on different days (50 g per day during 4 days of the week) or to divide the packages into smaller portions for daily consumption throughout the week. However, they were told not to consume more than 1 package (50 g) in one day. Additionally, to reduce bias related to omitting the consumption of the provided smoked salmon, participants filled out a daily control sheet to track their consumption of the intervention product. These records were monitored by the study researcher.

To assess the dietary intake of the participants at baseline, a three-day dietary record was used. Participants were instructed how to fill it in correctly, notably to note all foods and beverages they consumed during two weekdays (working days) and one weekend day (non-working day), while non-consecutive days characterized by a typical habitual diet were to be chosen, to specify the amounts using household measures, weight (g) or volume (mL), to include detailed information about all the products (e.g., fat % of dairy products, the type of bread, the kind of meat, the name of the shop or restaurant in case of consuming ready-to-eat meals, the brand of the beverage, etc.). When handing in the dietary records, each of them was controlled by the study researcher to make sure that they had been filled in correctly and that nothing had been omitted (e.g., snacks during the day). Special attention was placed on fats and oils, especially the ones added to the foods during meal preparation. In the case of any missing information, it was added at that stage, while the Polish Album of Photographs of Food Products and Dishes [29] was used to estimate the portion sizes in case of any uncertainties. The dietary intake analysis was conducted using Nuvero [30]—a professional dietetic program that uses the food product database from the Polish Food Composition Tables [31] and the US Department of Agriculture (USDA) database [32] for raw products with no data in the Polish database. The following diet characteristics were calculated: energy intake (kcal/day), total protein intake (g/day), animal protein intake (g/day), plant protein intake (g/day), animal protein/plant protein ratio in the diet, fat intake (g/day), saturated fatty acids (SFA) intake (g/day), monounsaturated fatty acids (MUFA) intake (g/day), polyunsaturated fatty acids (PUFA) intake (g/day), omega-6 fatty acids intake (g/day), omega-3 fatty acids intake (g/day), omega-6/omega-3 ratio in the diet, linoleic acid (LA) intake (g/day), alpha-linolenic acid (ALA) intake (g/day), eicosapentaenoic acid (EPA) intake (mg/day), docosapentaenoic acid (DPA) intake (mg/day), docosahexaenoic acid (DHA) intake (mg/day), cholesterol intake (g/day), carbohydrates intake (g/day), glucose intake (g/day), fructose intake (g/day), sucrose intake (g/day), lactose intake (g/day), fiber intake (g/day), as well as the energy percentage from protein, fats, and carbohydrates.

Aside from the intervention, study participants were instructed to maintain their regular diets, particularly concerning fish consumption. They were advised not to intentionally remove or add fish to their meals in ways that would alter their usual eating habits. Consequently, participants in the control group were free to eat any type of fish, including salmon, as long as it naturally fit into their routine diet. Similarly, those in the intervention group could include additional fish, beyond what was provided, salmon included, but only if it aligned with their established eating patterns.

### 2.4. Measurements

The study comprised two parts, each lasting four weeks, to evaluate participants’ status at the midpoint and after the full eight-week intervention. Lipid profiles were assessed three times: at baseline (before the intervention, w0), at midpoint (in week 5, w5), and after eight weeks (in week 9, w9). The anthropometric assessments were also conducted at baseline, at midpoint (week 5), and after eight weeks of intervention (week 9).

#### 2.4.1. Blood Test

For the lipid profile analysis, specialized nurses at the Warsaw University of Life Sciences Health Centre collected venous blood samples, which were then analyzed in Diagnostyka S.A.—a certified medical analysis laboratory in Warsaw. For the blood collection, the participants were asked to be in a fasting state (not to eat 12 h before the blood collection, while the last meal should not have been heavy or fatty) and not to drink alcohol 12 h before the measurement. All the blood draws were in the morning, between 7:40 and 9:00 a.m.

The lipid profile analysis included the measurement of total cholesterol (TC) concentration (mg/dL), high-density lipoprotein (HDL) concentration (mg/dL), low-density lipoprotein (LDL) concentration (mg/dL), triglycerides (TAG) concentration (mg/dL), while the non-HDL concentration (mg/dL) was calculated as the difference between TC and HDL. The recommended concentrations of the analyzed lipids and lipoproteins considered normal for individuals at low risk of CVD due to the young age of the participants [33], are presented in Table 2.

Based on the obtained lipid profile results, the following atherogenic indices were calculated: Atherogenic Index of Plasma (AIP), Atherogenic Coefficient (AC), Cholesterol Index (Cholindex), Castelli Risk Index 1 (CRI-1), Castelli Risk Index 2 (CRI-2) and the TAG/HDL ratio. The API was calculated as API = log(TAG/HDL) with TAG and HDL in molar concentrations [34], the AC as the ratio of non-HDL to HDL (ACI = non-HDL/HDL) [35], the Cholindex as the difference between LDL and HDL (Cholindex = LDL—HDL) with LDL and HDL in mg/dL concentrations [36], the CRI-1 as the ratio of TC to HDL (CRI-1 = TC/HDL) [37], and the CRI-2 as the ratio of LDL to HDL (CRI-2 = LDL/HDL) [37], and the TAG/HDL ratio was calculated using TAG and HDL concentrations expressed in mmol/L [38].

#### 2.4.2. Anthropometric Assessment

Participants were given specific instructions to prepare for the anthropometric assessment. They were asked to abstain from alcohol for 24 h before it, avoid vigorous exercise on the day of the assessment, refrain from drinking fluids for 3 h beforehand, and not eat for 4 h prior. Additionally, they were instructed to urinate just before the measurement, wear lightweight clothing without metal components (like a wireless sports bra), and remove any metal items (such as jewelry and watches). Each participant attended individually to maintain privacy, and their preparation was verified against these guidelines. Participants were also asked to remove bulky clothing, such as sweaters, and to take off their shoes and socks for the measurements.

First, weight and height were measured by a calibrated RADWAG WPT 200 OW scale-stadiometer (accuracy: 0.1 kg and 0.5 cm, range: 2–200 kg and 100–200 cm), and waist circumference (WC) and hip circumference (HC) were measured using a body measurement tape, while the waist was defined as the narrowest part of the torso, and the hip as the widest. The anthropometric measurements were performed according to commonly accepted protocols [39]. BMI was calculated using the Quetelet equation [23], and the waist-to-height (WHtR) and waist-to-hip (WHR) ratios were also calculated [40].

Body composition was measured using an AKERN BIA 101 Anniversary Physiological Data Analyzer, which uses a current of 50 kHz frequency and 400 µA intensity and 4 electrodes stuck on a person’s hand (2 electrodes) and foot (2 electrodes). The measurement was taken on the dominant (right or left) side of the participant’s body, while if a participant had metal implants in the body—on the side without these parts. The device displays the resistance (Rz) and the reactance (Xc), which were then, with the participant’s age, height, and weight, typed into the BodyGram program which allowed for the estimation of the sodium/potassium ratio (Na/K ratio), fat mass (FM), fat-free mass (FFM), body cell mass (BCM), body cell mass index (BCMI), muscle mass (MM), total body water (TBW), extracellular water (ECW), and intracellular water (ICW).

Participants’ results were compared and classified according to the following reference values:

- BMI of 25.0–29.9 kg/m^2^ was classified as overweight, while BMI ≥ 30.0 kg/m^2^ as obesity [23];
- WC of at least 88 cm was considered central obesity [7];
- WHtR of more than 0.5 was considered an additional risk factor for CVD [40];
- Body fat of below 36.0% was classified as normal, 36.0–41.9% as overweight, while of at least 42.0% as obesity [41].

To enhance adherence, immediately after the measurement, participants received only their weight and height results, whereas all body composition results were shared at the end of the study. The anthropometric measurements were conducted at the Dietetic Outpatient Clinic of the Department of Dietetics at Warsaw University of Life Sciences (WULS-SGGW).

Participants were instructed not to make any effort to lose weight during the study. However, some participants did experience weight loss (with a maximum of 5.0 kg during 8 weeks in the intervention group and 2.1 kg during 8 weeks in the control group). Despite this, there were no significant differences in body weight at baseline, mid-study, or after the intervention for the entire study population (*p* = 0.993), the intervention group (*p* = 0.960), or the control group (*p* = 0.987), as determined by Friedman’s test. At the same time, the proportion of participants who lost weight and those who did not lose weight in the intervention and the control group did not differ (in both groups: weight loss *n* = 10, no weight loss *n* = 9; *p* = 1.000, chi^2^ test).

### 2.5. Questionnaire

At baseline, all participants filled in an online questionnaire, prepared in GoogleForms, with questions concerning their socio-behavioral characteristics, as well as questions concerning fish, as follows:

- ‘What is your age?’—an open-ended question;
- ‘What is the highest level of education you have achieved?’—a close-ended question with three possible answers: completed secondary education (technical or general high school), completed undergraduate studies (holding a bachelor’s degree), or completed graduate studies (holding a master’s degree);
- ‘How would you evaluate your physical activity?’—a close-ended question with three possible answers: low (performing only essential activities), average (having some additional physical activity like walking or cycling), or high (practicing sports like team sports, gym, dance, fitness, running);
- ‘Do you like fatty fish (like salmon, herring, mackerel, sprats, and sardines)?’—a close-ended question with six possible answers: ‘I don’t like them at all’, ‘I don’t like them much’, ‘I’m indifferent about them’, ‘I quite like them’, ‘I like them a lot’, or ‘I don’t know’;
- ‘Do you think it’s true that consuming fish can help lower cholesterol levels?’—a close-ended question with three possible answers: ‘True’, ‘False’, or ‘I don’t know’;
- ‘Considering the last 6 months, how often have you consumed fish, fish products and other seafood (including sushi with fish or seafood)?’—a close-ended question with ten possible answers: ‘never’, ‘less than once a month’, ‘once a month’, ‘2–3 times a month’, ‘once a week’, ‘2 times a week’, ‘3–4 times a week’, ‘5–6 times a week’, ‘once a day’, or ‘more than once a day’. Due to the small number of responses in certain categories, for the group description, the answers were grouped into: ‘less than once a month’ (‘never’ and ‘less than once a month’), ‘one to three times a month’ (‘once a month’ and ‘2–3 times a month’), ‘once a week’, and ‘at least twice a week’ (‘2 times a week’, ‘3–4 times a week’, ‘5–6 times a week’, ‘once a day’, and ‘more than once a day’), which complies with fish intake recommendations [42,43,44].

Additionally, considering the fact that smoking seems to be associated with lipid profile [45] also in women [46], a question concerning the use of cigarettes was asked—a close-ended question with three possible answers: ‘I smoke traditional cigarettes’, ‘I smoke e-cigarettes’, or ‘I don’t smoke’. It was followed by an open-ended question concerning the number of cigarettes smoked in a week.

### 2.6. Study Course

**Figure 1:**
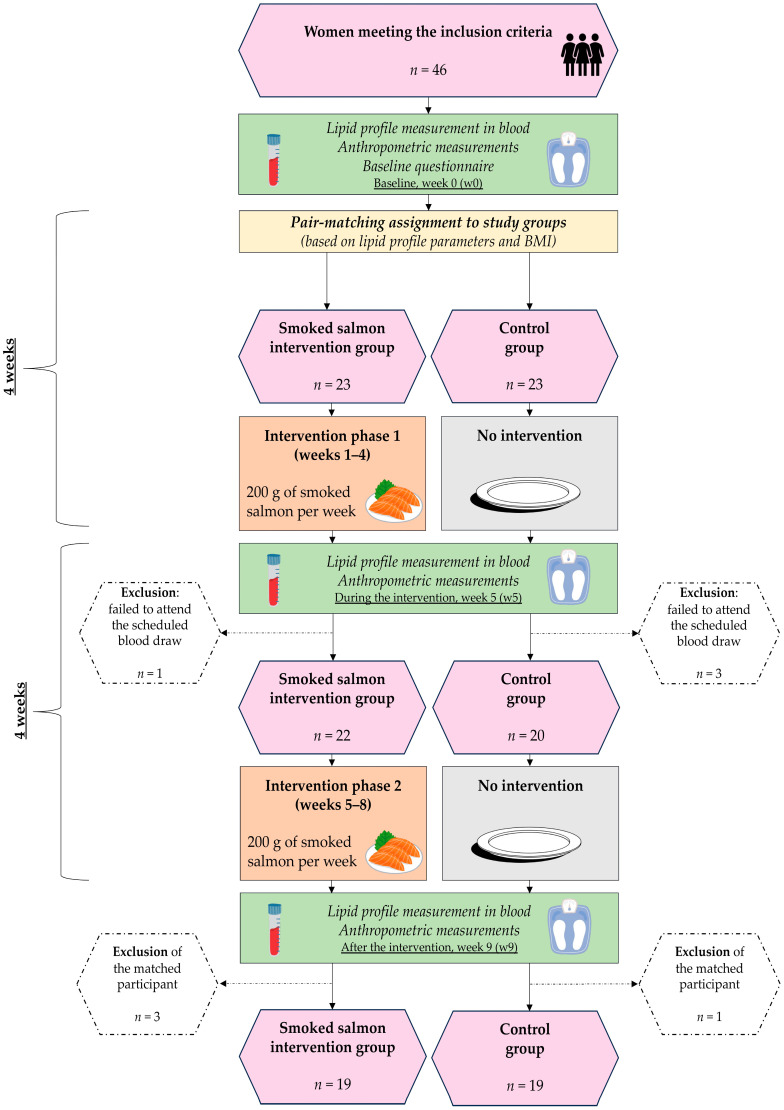
Study course. Lipid profile measurement in blood: total cholesterol (TC), high-density lipoprotein (HDL), non-HDL, low-density lipoprotein (LDL) and triglycerides (TAG). Anthropometric measurements: weight, height, waist circumference, hip circumference and body composition. Baseline questionnaire: age, education, physical activity, smoking cigarettes, preference toward fatty fish, opinion about cholesterol-lowering properties of fish, and habitual intake of fish.

The course of the conducted study is presented in Figure 1. Out of 57 women who expressed interest in participating in the study, 46 met the inclusion criteria and were randomly assigned to either the intervention or control group using a pair-matching method based on stratified block randomization, with stratification based on lipid profile (deviation from norms for TC, HDL, non-HDL, LDL, and TAG) and on BMI, resulting in 23 women in each group. The lipid profile parameters were either all within the recommended range (Table 2) for both paired participants, or some of the parameters were outside the recommended range for both. The BMI difference between the paired participants was not to exceed 10%. This ensured that the lipid profile parameters and BMI, as well as the number of participants with normal vs. elevated/low lipid profile components, were comparable between the groups at baseline (*p* > 0.05).

During the intervention, one participant from the intervention group and three participants from the control group were excluded due to failing to attend the blood draws in week 5. Therefore, after the study finished, their matched participants from the opposite group (three from the intervention group and one from the control group) were also excluded from the analysis. As a result, 38 participants (19 from the intervention group and 19 from the control group) were included in the final analyses.

### 2.7. Statistical Analyses

The threshold for statistical significance was set at *p* ≤ 0.05, while *p* ≤ 0.1 was regarded as indicative of a trend. The normality of distribution was assessed using the Shapiro–Wilk test.

The sample size required for the analyses was calculated based on the population of females in the studied age group living in the Masovian Voivodeship, where the study was conducted (318,200, according to the Central Statistical Office of Poland [47]). Assuming the frequency of excessive body mass (BMI ≥ 25.0 kg/m^2^) of 25.9% in this age group [48], the target population was determined as 82,414. A 90% confidence level, 15% margin of error, and 60% dyslipidemia prevalence in the population of overweight/obese individuals [49] were applied, resulting in an estimated required sample size of 29. The final sample of 38 females was therefore considered sufficient.

To compare the groups, when data were normally distributed and met the homogeneity of variance assumption (as determined by Levene’s test, *p* > 0.05), the Student’s *t*-test was applied, while when data were non-normally distributed or normally distributed but did not meet the homogeneity of variance assumption (as determined by Levene’s test, *p* ≤ 0.05), the U Mann–Whitney test was applied. For analyses of differences between the groups regarding categorical variables, the chi-square (chi^2^) test with Yates’ continuity correction was applied.

For more in-depth analyses, the study participants were divided into subgroups based on their BMI category: overweight (BMI 25.0–29.9 kg/m^2^) and obese (BMI ≥ 30.0 kg/m^2^) [23] and based on their WHtR: lower than 0.5 (no additional CVD risk factor) and higher than 0.5 (additional CVD risk factor) [40].

All analyses were conducted using Jamovi (The Jamovi project, version 2.63, Sydney, Australia) [50] software.

## 3. Results

### 3.1. Baseline Characteristics of the Studied Group

The socio-behavioral characteristics of the study participants concerning education, physical activity, and smoking cigarettes are presented in Table 3. Almost half of the participants completed their secondary education, one-third held a bachelor’s degree, and one-fifth held a master’s degree. Most of them rated their physical activity as average, one-fourth as low, and one-fifth as high. No differences were noted in the physical activity levels between the groups—4 participants (21%) from the intervention group and 5 participants (26%) from the control group assessed it as low, 11 participants (58%) from the intervention group and 10 participants (53%) from the control group assessed it as average, and 4 participants (21%) from the intervention group and 4 participants (21%) from the control group assessed it as high (*p* = 0.924, chi^2^ test). One in ten declared to smoke traditional cigarettes, two women declared to smoke e-cigarettes, while more than 80% declared not to smoke.

Table 4 presents the participants’ answers concerning their preference toward fatty fish, their opinion about the cholesterol-lowering properties of fish, and the participants’ habitual fish intake at baseline. Most of the participants declared to like fatty fish a lot, one-fifth to quite like them, and 5% to be indifferent about them. More than half believed that eating fish helps decrease cholesterol levels; 5% did not think that, while 42% declared not to know. The majority of them declared to have consumed fish, fish products, and other seafood one to three times a month in the last 6 months, 18.5% once a week, also 18.5% at least twice a week, and one woman declared to have consumed them less frequently than once a month.

Table 5 presents the detailed anthropometric characteristics of the intervention and the control group at baseline, as well as the weight change from baseline to after the intervention (w0 to w9). There were no significant differences in all anthropometric characteristics of the participants between the two studied groups (*p* > 0.05). The BMI of 15 participants (79%) in the control group and 14 (74%) in the intervention group corresponded to overweight, and of 4 participants (21%) in the control group and 5 (26%) in the intervention group to obesity, and it did not differ between the two groups (*p* = 1.000, chi^2^ test). Five participants (26%) in each group had a waist circumference of at least 88 cm (data not presented in the table). The conducted analysis confirmed that the intervention and control group were comparable at baseline.

The body composition of the participants from the intervention and the control group at baseline is presented in Table 6. All of the analyzed body composition characteristics were the same across the two groups (*p* > 0.05). Considering the alternative recommendations to classify overweight and obesity based on body fat mass percentage, instead of BMI, the body fat of 17 participants (45%) was at least 36% but less than 42%, which is classified as overweight, the body fat of 13 participants (34%) was at least 42%, which is classified as obesity, while the body fat of 8 participants (21%) was below 36%, which is classified as normal (data not presented in the table).

The energy value and macronutrient intake in the diets of the participants from the intervention and the control group at baseline are presented in Table 7. The diet characteristics did not differ across the groups (*p* > 0.05).

### 3.2. Intervention Efficacy

The lipid profile and the calculated atherogenic indices of the participants from the intervention and the control group at baseline, during the intervention (after 4 weeks of intervention, in week 5), and after the intervention (in week 9) are presented in Table 8. At baseline, the median TC concentration amounted to 171.9 mg/dL in the intervention group and 174.7 mg/dL in the control group; the HDL to 58.1 mg/dL and 55.9 mg/dL, the non-HDL to 124.2 mg/dL and 112.9 mg/dL, the LDL to 101.4 mg/dL and 89.3 mg/dL, and the TAG to 89.9 mg/dL and 99.4 mg/dL, in the intervention and in the control group, respectively. The concentrations did not differ between the two groups at baseline (*p* > 0.05). Surprisingly, they did not differ during and after the intervention, either (*p* > 0.05). The calculated atherogenic indices did not differ across the groups either at baseline or during and after the intervention (*p* > 0.05).

The lipid profile changes from baseline to the middle of the intervention (from w0 to w5), from the middle to after the intervention (from w5 to w9), and from baseline to after the intervention (from w0 to w9) are presented in Table 9. While no statistically significant differences were observed between the two groups concerning the first half of the intervention (from w0 to w5; *p* > 0.05), a trend was noted for the LDL concentration change in the second half of the intervention (from w5 to w9). In the intervention group, it decreased with a mean of 5.5 ± 21.4 mg/dL, whereas in the control group, it increased with a mean of 6.8 ± 21.7 mg/dL (*p* = 0.087). Concerning the whole intervention period, namely from w0 to w9, a statistically significant difference was observed concerning the LDL, which decreased on average by 8.2 ± 20.7 mg/dL in the intervention group and increased on average by 9.5 ± 20.0 mg/dL in the control group (*p* = 0.011). Moreover, a non-significant but very close to statistical significance (*p* = 0.053) difference was noted for the TAG concentration change from w0 to w9. A median increase of 17.0 mg/dL was observed in the intervention group, while a median decrease of 7.3 mg/dL was seen in the control group. These results indicate that the intervention was mostly effective concerning the LDL concentrations. However, what should also be noted is the decreasing *p*-value in almost all analyzed lipid metabolites (e.g., TC change: from w0 to w5 *p* = 0.645, from w5 to w9 *p* = 0.283, and from w0 to w9 *p* = 0.199). This might indicate that had the intervention lasted longer, differences concerning the other lipid metabolites may have also been observed.

Table 10 presents the lipid profile changes from baseline to the middle of the intervention (from w0 to w5), from the middle to after the intervention (from w5 to w9), and from baseline to after the intervention (from w0 to w9) across overweight participants (BMI 25.0–29.9 kg/m^2^). Similar to when all participants were included in the analysis, the observed difference between the two groups was noted for the LDL change from w0 to w9. A mean decrease of 3.6 ± 19.1 mg/dL was observed in the intervention group, while an increase of 12.6 ± 20.7 mg/dL was noted in the control group (*p* = 0.038). Interestingly, a trend of differences was seen in the change of non-HDL from w0 to w9 in this analysis. While a mean increase of 11.6 ± 18.3 mg/dL was noted in the control group, in the intervention group, it was almost constant (0.1 ± 17.3 mg/dL; *p* = 0.095).

In the subgroup of obese participants (BMI ≥ 30.0 kg/m^2^), no differences were noted between the intervention and the control group (*p* > 0.05), probably due to the small number of participants (*n* = 9; 5/4—intervention/control group) (Supplementary Materials Table S1).

The lipid profile changes from baseline to the middle of the intervention (from w0 to w5), from the middle to the end of the intervention (from w5 to w9), and from baseline to after the intervention (from w0 to w9) across participants with a WHtR > 0.5 are presented in Table 11. In this subgroup, differences concerning the LDL and TAG change from baseline to after the intervention (w0 to w9) were noted. Regarding the LDL change, in the intervention group, a mean decrease of 12.8 ± 21.8 mg/dL was observed, while in the control group, an increase of 7.7 ± 18.1 mg/dL was indicated (*p* = 0.027), similar to when all participants were taken into the analysis. Additionally, the TAG change, which showed a non-significant but very close to statistical significance difference when all participants were taken into consideration, showed a statistically significant difference (*p* = 0.040) across participants with a WHtR > 0.5 with, again, an increase of 50.0 ± 68.3 mg/dL in the intervention group and a decrease of 8.6 ± 56.6 mg/dL in the control group.

In the subgroup of participants whose WHtR was lower than 0.5 (*n* = 16; 8/8—intervention/control group), which is considered normal, there were no statistically significant differences between the lipid profile changes across the two groups (*p* > 0.05) (Supplementary Materials Table S2). These results indicate that among participants with a greater than 0.5 WHtR, the intervention was associated with a positive from a health perspective decrease in LDL and a negative from a health perspective increase in TAG.

The number of participants with lipid profile components considered as normal vs. elevated/low at baseline, during, and after the intervention is presented in Table 12. While no differences across the two studied groups were observed either at baseline or during and after the intervention (*p* > 0.05), what should be noted is that at baseline, 32% of all participants had elevated total cholesterol concentrations, 16% had low HDL concentrations, 24% had elevated non-HDL concentrations, 29% had elevated LDL concentrations, and 16% had elevated TAG concentrations. Notably, at baseline, 18 of all participants (47%) had at least one lipid profile parameter outside the recommended range (data not presented in the table).

Table 13 presents the number of participants for whom a positive change was observed considering a decrease (of TC, non-HDL, LDL, TAG) or an increase (of HDL) in the concentration of the specific lipid profile components from baseline to after 8 weeks of the intervention. Importantly, a trend of differences was noted for the total cholesterol change. On a level close to significance, more participants from the intervention group experienced a decrease (a positive change) in the concentration of total cholesterol after 8 weeks of the intervention compared to the participants from the control group (*p* = 0.100). No differences were noted for the other lipid profile components (*p* > 0.05).

The changes in the analyzed atherogenic indices during the study (from baseline to after the intervention, w0 to w9) are presented in Table 14. A difference across the intervention and the control group was seen for the Cholindex. While a median decrease of 4.4 mg/dL was noted in the intervention group, in the control group, a median increase of 0.8 mg/dL was observed (*p* = 0.040). Additionally, trends of differences were noted for the change of the AIP index, the CRI-2 index, and the TAG/HDL ratio (*p* = 0.054, *p* = 0.099, *p* = 0.057, respectively).

The number of participants whose indices decreased vs. those whose indices were stable or increased during the study between the two groups was also analyzed for all the indices separately. However, no differences were noted for any of the indices (*p* > 0.05, chi^2^ test) (data not presented in the table).

## 4. Discussion

### 4.1. Participants’ Preference Toward Fatty Fish, Opinion About Cholesterol-Lowering Properties of Fish, and Habitual Fish Intake at Baseline

Almost three in four participants indicated liking fatty fish (such as salmon, herring, mackerel, sprats, and sardines) a lot, while one-fifth declared to quite like them. Only 5% indicated to be indifferent about them, while none stated not to like them. Given that the average intake of seafood-derived omega-3 fatty acids in Poland is below the recommended levels [17], as well as that fatty fish are recommended as part of a healthy diet [42,43,44], especially for people at higher cardiovascular risk [12] this seems to be a promising result. However, the method of gathering the study participants was convenience sampling; therefore, these results cannot be seen as representative. Interestingly, more than half of the participants believed that consuming fish can help lower cholesterol levels, which is true according to the meta-analysis by Alhassan et al. [21]. However, what seems unexpected considering the declarations of liking fatty fish described above, only 18.5% of participants comply with the recommendations to consume fish at least twice a week [42,43,44]. Most of them declared to consume fish only one to three times a month. This indicates that despite a high preference for fatty fish, these young Polish women do not consume them on a regular basis.

### 4.2. Cardiovascular Risk Characteristics of the Study Participants

The results from the presented study indicate that the problem of dyslipidemia occurs among overweight and obese women despite their young age (18–30 years). Depending on the specific lipid profile parameters, between 16 and 32% of all participants had elevated TC, LDL, non-HDL or TAG, or low HDL before the intervention began. However, what is even more alarming, almost every second participant had at least one parameter of the lipid profile outside the recommended range. This problem is also observed among even younger people worldwide. In the USA, 25% of adolescents aged 12–19 have at least one adverse level of TC, LDL, HDL, non-HDL, TAG, or apolipoprotein B [51]. In a recently published study conducted among Chinese college students, 13% of them were found to have dyslipidemia [52], while its prevalence among Saudi Arabia adolescents aged 10–19 accounts for 25% [53]. The rate of dyslipidemia in the presented study (47%) is higher than in the cities studies [51,52,53], probably due to the fact that it considered only participants with overweight or obesity, contrary to the cited cross-sectional studies in which participants with normal body mass and underweight were also included [51,52,53].

Almost one-third of the study participants were classified as central obese based on their waist circumference [7], while more than half of them had a waist-to-height ratio above 0.5. According to a meta-analysis by Xue at al. [40], both increased WC and WHtR were found to increase the risk of cardiovascular disease, which underlines how important it is to search for interventions aiming at decreasing that risk. Moreover, one-fourth of the participants declared to have low physical activity, which is alarming, as physical activity is known to have a positive effect on managing cardiovascular disease risk [7]. According to a study by Huffman et al. [54], a rigid, supervised exercise intervention resulted in beneficial effects on lipid profile characteristics regardless of adhering to the diet recommended by the AHA [54].

### 4.3. Intervention Efficacy in Improving Lipid Profile Components

While no differences between the intervention and the control group were noted considering the absolute values of the concentrations of the different lipid profile parameters (TC, HDL, non-HDL, LDL, TAG) either during or after the intervention, differences considering the concentration changes were observed. After 8 weeks of consuming 200 g of farmed smoked salmon weekly, participants from the intervention group experienced a decrease in the LDL concentration, while during the same period, those from the control group experienced an increase. A recent meta-analysis by Hasan et al. [6] concluded that a lifestyle intervention resulting in a loss of 1 kg of body weight reduces the LDL by 1.28 mg/dL (95% CI, −2.19 to −0.37 mg/dL). In the presented study, incorporating 200 g of smoked salmon into a weekly diet resulted in a more than sixfold reduction in LDL, which is a very promising result in favor of increasing fatty fish intake.

The meta-analysis by Alhassan et al. [21] indicated that moderate consumption of 20–150 g of oily fish per day is associated with statistically significant decreases in TAG and increases in HDL in comparison with a control group [21]. Although in the presented study, the salmon fish consumption corresponded to 28.6 g of oily fish per day, which is in the range stated to be effective in the meta-analysis by Alhassan et al. [21], the results were markedly different. In the presented study, no significant differences concerning the HDL and TAG were observed, whereas the meta-analysis by Alhassan et al. [21] did not show significant effects of oily fish consumption on LDL concentration change, contrary to the presented study. A more recent review of intervention studies stated that while some fish-based interventions resulted in decreasing TAG, other studies did not report any significant effects concerning the TAG, especially when the TAG was not elevated at baseline [55].

Interestingly, no differences between the two groups were seen during the first 4 weeks of the intervention; only a trend of differences was noted for the w5 to w9 change of the LDL, and a statistically significant difference was noted only for the w0 to w9 change. This indicates that there might be more time needed to observe any changes in the lipid profile. On the other hand, a crossover intervention study among normolipidemic to mildly hyperlipidemic but apparently healthy men and women aged 23–65 in the USA, in which one of the intervention groups consumed around 226 g of salmon weekly, resulted in decreases in TAG and increases in HDL compared to the control group only after 4 weeks, indicating that even such a short time might be sufficient to observe statistically significant changes [56]. Such different results are probably due to the fact that the cited study [56] was a controlled feeding trial in which the diets did not include any omega-3-fatty-acid-rich foods other than those being tested (even concerning the use of oils, only olive oil and corn oil were allowed as added fats), whereas the presented study assumed a more real-life context, in which the participants in the control group were instructed not to alter their usual eating habits; therefore, they were allowed to consume fish and other omega-3 sources. Additionally, the cited study [56] was published in 2009, which might indicate that the salmon used in it might have had a significantly different nutritional value compared to the salmon used in the present study (as in recent years, the diets given to farmed salmon have changed resulting in changed nutritional value [22]); however, no detailed information about the salmon used in it was provided. Moreover, the older age of the participants in the cited study [56] might have played a role, too.

Concerning the various atherogenic indices analyzed in the presented study, differences across the two studied groups were seen for the Cholindex change during the study. A positive from a health perspective decrease was noted in the intervention group, while a negative from a health perspective increase was observed in the control group. These differences correspond to the fact that differences concerning the LDL changes across the groups were indicated, as the LDL concentration is used in the formula to calculate Cholindex. Nevertheless, a decrease in that index is very positive, as it is considered to be more strongly associated with CVD risk than other lipid parameters considered individually [36].

The results of the presented study indicate that the intervention was mostly effective concerning the LDL concentrations and, consequently, the changes in the Cholindex. However, what should also be noted is the decreasing *p*-value in almost all analyzed lipid metabolites. This might indicate that had the intervention lasted longer, differences concerning the other lipid metabolites may have also been observed.

A negative change observed in the presented study was the increase in TAG in the intervention group compared to the control group. While for the whole studied group, the difference was very close to statistically significant, for participants of higher cardiovascular risk (WHtR > 0.5), the difference was statistically significant, and an increase was noted in the intervention group and a decrease in the control group. Contrary to these results, results from a 4-week intervention study in which overweight men and women consumed farmed Atlantic salmon twice a week with different portion sizes ranging from 90 to 270 g indicated that even at the lowest salmon intakes, namely 180 g weekly, decreases in TAG were observed [57]. Additionally, the previously cited meta-analysis by Alhassan et al. [21], as well as a more recent review of randomized control trials [55], stated that consuming oily fish seems to have a TAG-lowering effect in both healthy and non-healthy populations. However, the latter review [55] also indicated lipidomics studies in which increases in some of the TAGs (usually those with a longer chain and a greater number of desaturations such as TAG(22:6) and TAG(20:5) or TAG(56:5) and TAG(56:7)) were associated with increasing fatty fish intake, suggesting that fish intake does not only influence TAG concentrations but possibly also remodels the structure. It concluded that determining the optimal quantity of fish needed to effectively reduce TAG remains challenging [55].

The changes in the lipid profile parameters observed in the presented study, namely the decrease in LDL concentration and the TAG concentration increase in the intervention group, can be most probably attributed to the long-chain omega-3 fatty acids found in salmon, such as the ALA, EPA, and DHA. The presence of EPA and DHA, primarily found in fish and seafood [18], is the reason why fish are recommended in various nutritional guidelines [25], including the ones from the AHA, to reduce cardiovascular risk [12,16]. However, in intervention studies using purified EPA and DHA, EPA was found not to influence LDL concentrations, while the DHA increased its concentrations [58], contrary to the results of the presented study. On the other hand, replacing SFA with MUFA was seen to lower LDL concentration in moderately hypercholesterolemic individuals [59], while replacing the intake of oil high in LA and low in ALA with oils high in MUFA and ALA and low in LA resulted in LDL concentration decreases [60]. Because in the presented study MUFA accounted for 53%, while omega-3 fatty acids accounted for 14% of the whole fat in the intervention salmon, it might somehow explain the results. On the other hand, the omega-6 content was as high as omega-3 and also accounted for 14%. Contrary to the cited studies [59,60], a meta-analysis by Cao et al. [61] indicated that the consumption of MUFA does not affect LDL concentrations but can lower TAG. Moreover, as described above, other fish-based intervention studies [56,57] and meta-analyses [21,55] found that increasing oily fish intake reduces TAG and increases HDL, which was not observed in the presented study.

### 4.4. Content of Various Fatty Acids in Salmon

Although salmon belongs to the group of fatty fish, which are known to be good sources of omega-3 fatty acids [62], it seems that the differences between various salmon samples are huge, which can be seen when comparing the nutritional value of 100 g of the smoked salmon used in the intervention with data for smoked salmon from the USDA [63]. In the presented study, the MUFA corresponded to 5.23 g, while in the USDA database, to 2.02 g; the ALA (C18:3n3) in the presented study was 450 mg, while PUFA 18:3 in USDA is 0 mg; the EPA in the presented study was 290 mg, in the USDA database 183 mg; the DHA in the presented study was 320 mg, while in the USDA database 267 mg [63]. In the presented study, the total lipids corresponded to 9.87 g and in the USDA database to 4.32 g, which is also associated with the energy value of the smoked salmon—in the presented study 176 kcal and in the USDA database 117 kcal per 100 g [63]. The smoked salmon used in the intervention was of farmed origin, while the one in the USDA database may have been, as it is not stated there, of wild origin. An analysis of the different fatty acids in salmon of various origins [27] confirms this difference. While the omega-6/omega-3 ratio in farmed salmon in that analysis was 0.7 ± 0.01, similar to 0.9 in the salmon used in the intervention, in wild salmon, it was 0.05 ± 0.01, which is more than ten times less [27].

A more profound insight can be provided when analyzing the fatty acid composition of raw farmed Atlantic salmon according to the USDA database [64]. According to that analysis, the EPA in the salmon was 862 mg compared to 290 mg in the salmon used in the intervention, and the DHA 1100 mg compared to 320 mg [64]. In both cases, the content of the beneficial fatty acids (EPA and DHA) is around three times more in the salmon in the USDA table than in the salmon used in the intervention. Importantly, these data were not updated after 2008 [64], which might indicate that the nutritional value of farmed Atlantic salmon has changed substantially in recent years, with less EPA and DHA at present. This seems to be reflected in intervention studies conducted using farmed Atlantic salmon. In the presented study, which was conducted in 2021–2022, the 200 g of smoked salmon weekly provided 174 mg of EPA + DHA daily, while in a study of a similar design but conducted ten years before (2010–2012) [57], 180 g of farmed Atlantic salmon weekly provided 307 mg of these fatty acids, which is 1.8 times more than in the presented study. These differences might be due to the different feed provided to the salmon as well as the fact that the cited study was conducted in the USA [57], while the presented one was in Poland. Nevertheless, the differences in the EPA + DHA content in various salmon samples, even across farmed Atlantic salmon, are clear.

One might wonder whether the process of smoking does not decrease the amount of the beneficial EPA and DHA. However, studies show that the EPA and DHA concentrations in Atlantic salmon after cold smoking are not different than in raw salmon, while the process of smoking has a protective effect on the EPA and DHA, with a three times higher loss of these fatty acids in raw samples compared to smoked samples during storage [65].

A small cross-over study conducted in a group of 20 clinically healthy adults showed very interesting and thought-provoking results concerning the influence of the content of EPA + DHA and omega-6/omega-3 on lipid profile [66]. Participants in that study consumed 630 g of gilthead sea bream weekly, which was fed with either 100% fishmeal or fishmeal mixed with plant proteins (50%/50%). While the intake of EPA + DHA was similar in both groups, the omega-6/omega-3 ratio was almost two times higher in the group consuming gilthead sea bream fed with fishmeal mixed with plant protein compared to fed with 100% fishmeal (0.91 vs. 0.48). Importantly, the lipid profile of the study participants was influenced greatly by the diet they consumed. In the group that first consumed the fish fed with 100% fishmeal, significant decreases in TC, LDL, and TAG were seen after that phase, while after the second phase (consuming fish fed with fishmeal mixed with plant protein), their concentrations returned to baseline levels. In the other group, no differences were seen after their first phase (consuming fish fed with fishmeal mixed with plant protein), while an increase in TC was noted after the second phase of the intervention (consuming the fish fed with 100% fishmeal) [66].

These compelling findings, dating back to 2013, clearly suggest that not only the EPA + DHA sum but possibly even more so the omega-6/omega-3 ratio in the consumed fish, determines the various changes in lipid profile in terms of primary prevention of CVD. Of note, as written above, despite consuming 630 g of gilthead sea bream fed with fishmeal mixed with plant protein weekly and subsequently high intakes of EPA + DHA, no changes were observed concerning the lipid profile parameters of the participants [66]. This might help explain the results of the presented study, in which no differences were seen for the TC and TAG across the groups, as the omega-6/omega-3 ratio of the salmon used in the presented intervention was 0.9—just like in the cited study [66]. Despite conflicting results, it seems that higher omega-6/omega-3 ratios in the blood are connected to negative cardiovascular outcomes [67]; therefore, the omega-6/omega-3 ratio in the salmon consumed seems to play a role in providing health benefits.

Over the past two decades, commercial diets of farmed salmon have changed toward a more plant-based diet due to the limited availability of marine fishmeal and fish oil, which is known to result in decreased EPA + DHA and increased omega-6 fatty acids [22]. According to Sissener [22], while numerous dietary interventions with salmon showed beneficial effects on fatty acids profiles and health parameters in humans, many of these studies used salmon fed with high levels of marine-derived feed. The results from the presented study, in which, looking at the fatty acids composition of the used salmon, the salmon were probably fed with plant-based ingredients, seem to indicate that the beneficial effects of increasing salmon intake on various health outcomes seen in meta-analyses [13,14,68,69,70,71,72] might not necessarily be noted in studies conducted at present due to a less beneficial omega-6/omega-3 ratio.

However, what should also be stated is that salmon is not only a source of essential fatty acids in the human diet [16], but it can also provide significant amounts of vitamin D, which is especially important when no skin synthesis is possible due to the latitude of the place of residence and the season of the year [73]. Vitamin D3 content in commercially available farmed Atlantic salmon in Poland is stated to be 21.3 μg/100 g [62], which covers 142% of the recommended adequate intake of 15 μg daily [74]. Salmon can provide selenium and iodine to the human diet, as well [31].

### 4.5. Strengths and Limitations

The presented study is, to the best of our knowledge, the first to assess the influence of increasing farmed salmon intake on lipid profile characteristics in young women with excessive body weight without providing a weight-loss diet. Moreover, the amount of salmon used in the intervention complied with fish intake recommendations and can be easily incorporated into a person’s habitual diet, making it possible to adhere to such a diet in a real-life context. Importantly, despite only a modest dietary adjustment of incorporating 200 g of smoked salmon weekly into the participants’ diet, significant differences between the two studied groups were seen for the LDL change, especially in participants with a WHtR ratio of more than 0.5, as well as for the Cholindex, which is an important atherogenic index. These noteworthy results indicate that increasing fatty fish intake, such as the intake of salmon, might be one of the recommendations that could be given to young women with excessive body weight who are at risk of developing cardiovascular disease in the future.

Despite numerous strengths, the study also has limitations. The study ultimately included only 38 participants, which limits the ability to generalize the findings to larger populations. Additionally, since some of the analyzed parameters had non-significant but close-to-statistically-significant differences, a greater sample size might have resulted in more statistically significant differences across the studied group. However, as the study assumed the inclusion only of young women with excessive body weight residing in Warsaw or its surroundings (due to the multiple visits to the place of conducting the study), gathering a larger group for the study was not feasible. Moreover, since the intervention lasted only 8 weeks, the long-term effects of increasing farmed salmon intake on the analyzed parameters, as well as the real CVD risk reduction, cannot be assessed. Additionally, considering the observed TAG increase in the intervention group compared to a decrease in the control group among participants with a WHtR of more than 0.5, as well as the fact that TAG concentrations can be influenced by other lifestyle factors, such as alcohol consumption, it might have been beneficial to assess participants’ alcohol intake throughout the study. However, while some studies show that higher alcohol consumption is associated with higher TAG concentrations [75], others indicate that the incidence of elevated TAG is lowest in subjects with moderate alcohol consumption (10–20 g/alcohol a day), similar to the J-shaped association between alcohol consumption and cardiovascular risk [76]; hence, it seems that, to date, the question if and how alcohol consumption influences TAG concentrations is still unsolved. Last but not least, since physical activity may influence lipid metabolism [77], it might have been beneficial to assess participants’ physical activity throughout the study time. However, at baseline, no differences were noted in the physical activity level between the groups. At the same time, participants were clearly instructed not to make efforts to lose weight, which can be achieved through an increase in physical activity or diet alterations, which was also restricted during the intervention period.

## 5. Conclusions

Despite increasing the intake of farmed smoked salmon to 200 g weekly, no differences were observed in the absolute values of the analyzed lipid profile parameters and atherogenic indices between the intervention and the control group after the 8-week-long intervention. However, differences in certain changes were noted, such as for the LDL and the Cholindex), for which decreases were noted in the intervention group, while increases were seen in the control group. On the other hand, across participants with WHtR > 0.5, an increase in TAG in the intervention group and a decrease in the control group was noted. Regarding the observed beneficial influence of increasing salmon intake to the recommended amounts on decreasing LDL and Cholindex in young women with excessive body weight after 8 weeks, it seems that such a diet alteration might be recommended for this group to decrease their risk of CVD in the future. Nevertheless, regarding the diverse influence on TAG, further studies are needed to assess the long-term influence of increasing the intake of fatty fish available on the market at present on human health.

## Figures and Tables

**Table 1:** Nutritional value of the smoked salmon used in the intervention per 100 g.

	100 g of Smoked Salmon
Energy, kcal	176
Fat, g	9.87
SFA, g	1.70
MUFA, g	5.23
PUFA, g	2.94
Omega-6 fatty acids, mg	1270
Omega-3 fatty acids, mg	1410
Omega-6/omega-3 ratio	0.9
ALA (C18:3n3), mg	450
EPA (C20:5n3), mg	290
DHA (C22:6n3), mg	320
EPA + DHA, mg	610
Trans fatty acids, g	<0.1
Protein, g	20.12
Salt, g	1.67

SFA—saturated fatty acids; MUFA—monounsaturated fatty acids; PUFA—polyunsaturated fatty acids; Omega-6 fatty acids—the sum of omega-6 fatty acids; Omega-3 fatty acids—the sum of omega-3 fatty acids; ALA—alpha-linolenic acid; EPA—eicosapentaenoic acid; DHA—docosahexaenoic acid; EPA + DHA—the sum of eicosapentaenoic and docosahexaenoic acid.

**Table 2:** Recommended concentrations of the analyzed lipids and lipoproteins considered normal in the analyses [33].

	Recommended Concentration, mg/dL
TC	<190.0
HDL	>45.0
Non-HDL	<145.0
LDL	<115.0
TAG	<150.0

TC—total cholesterol, HDL—high-density lipoprotein, LDL—low-density lipoprotein, TAG—triglycerides.

**Table 3:** Socio-behavioral characteristics of the study participants concerning education, physical activity, and smoking cigarettes.

Socio-Behavioral Characteristics		Number of Participants (%)
Education	Completed secondary education (technical or general high school)	18 (47%)
	Completed undergraduate studies (holding a bachelor’s degree)	12 (32%)
	Completed graduate studies (holding a master’s degree)	8 (21%)
Physical activity	Low	9 (24%)
	Average	21 (55%)
	High	8 (21%)
Smoking cigarettes	Traditional cigarettes	4 (11%)
	E-cigarettes	2 (5%)
	Not smoking	32 (84%)

**Table 4:** Participants’ answers concerning their preference toward fatty fish, their opinion about cholesterol-lowering properties of fish, and the participants’ habitual fish intake at baseline.

Question Concerning Fish	Answer	Number of Participants (%)
Fatty fish preference	Likes a lot	28 (74%)
	Quite likes	8 (21%)
	Indifferent	2 (5%)
	Does not like much	0 (0%)
	Does not like at all	0 (0%)
	Does not know	0 (0%)
Agreement with the statement that consuming fish can help lower cholesterol levels	True	20 (53%)
	False	2 (5%)
	I don’t know	16 (42%)
Frequency of consuming fish, fish products, and other seafood (including sushi with fish or seafood) in the last 6 months	Less than once a month	1 (3%)
	One to three times a month	23 (61%)
	Once a week	7 (18.5%)
	At least twice a week	7 (18.5%)

**Table 5:** Anthropometric characteristics of the intervention and the control group at baseline, as well as the weight change throughout the study time.

Anthropometric Characteristic	Intervention Group (*n* = 19)		Control Group (*n* = 19)		p **
	Mean ± SD	Median (Min–Max)	Mean ± SD	Median (Min–Max)	
Age, years	23.1 ± 3.7	22.0 (18.0–29.0)	22.9 ± 3.5	23.0 (19.0–30.0)	0.859
Weight, kg	80.2 ± 11.1	75.6 (66.2–110.8) *	80.1 ± 9.1	78.2 (67.2–102.5)	0.693
Height, cm	166.6 ± 5.4	167.0 (156.5–173.5)	167.6 ± 5.7	166.0 (157.5–177.0)	0.583
WHtR	0.51 ± 0.04	0.50 (0.46–0.61)	0.51 ± 0.04	0.50 (0.45–0.59)	0.557
BMI, kg/m^2^	28.8 ± 3.1	27.9 (25.5–36.8) *	28.5 ± 2.8	27.9 (25.3–34.5) *	0.885
Waist circumference, cm	85.7 ± 7.1	84.1 (78.0–106.3) *	84.9 ± 6.8	83.5 (75.5–98.2)	0.759
Hip circumference, cm	111.2 ± 6.8	109.8 (102.2–126.5)	112.4 ± 5.1	112.2 (104.8–124.5)	0.532
WHR	0.77 ± 0.03	0.78 (0.72–0.84)	0.75 ± 0.04	0.75 (0.67–0.84)	0.160
Weight change during intervention, kg	0.4 ± 3.4	−0.4 (−5.0–7.7)	0.3 ± 1.18	−0.1 (−2.1–4.2)	0.942

* Non-normal distribution (verified using Shapiro–Wilk test; *p* ≤ 0.05); ** Student’s *t*-test or U Mann–Whitney test (depending on data distribution); WHtR—waist-to-height ratio; BMI—body mass index; WHR—waist-to-hip ratio.

**Table 6:** Body composition of participants from the intervention and the control group at baseline.

Body Composition Characteristic	Intervention Group (*n* = 19)		Control Group (*n* = 19)		p **
	Mean ± SD	Median (Min–Max)	Mean ± SD	Median (Min–Max)	
Na/K ratio	0.9 ± 0.1	0.9 (0.8–1.0) *	0.9 ± 0.1	0.9 (0.8–1.0) *	1.000
FM, kg	31.7 ± 7.9	29.6 (23.9–52.8) *	32.6 ± 5.9	31.4 (24.4–46.2)	0.385
FM, %	39.2 ± 4.7	37.6 (32.8–47.7)	40.5 ± 3.7	40.5 (34.6–48.8)	0.327
FFM, kg	48.4 ± 4.7	48.5 (40.7–58.0)	47.5 ± 4.7	45.4 (40.1–56.8)	0.538
FFM, %	60.8 ± 4.7	62.4 (52.4–67.2)	59.5 ± 3.7	59.5 (51.2–65.4)	0.327
BCM, kg	25.3 ± 3.3	25.4 (19.0–31.0)	24.4 ± 3.1	24.4 (20.4–29.5)	0.422
BCM, %	31.7 ± 3.3	32.1 (26.0–36.8)	30.5 ± 2.6	31.5 (24.4–34.3)	0.248
BCMI, kg/m^2^	9.1 ± 0.9	9.2 (7.3–10.4)	8.7 ± 0.8	8.5 (7.5–10.3)	0.145
MM, kg	31.2 ± 3.9	31.5 (24.0–38.1)	30.2 ± 3.6	29.9 (25.4–36.2)	0.433
MM, %	39.1 ± 3.8	39.8 (32.6–44.9)	37.8 ± 3.0	38.6 (30.5–42.1)	0.251
TBW, L	35.5 ± 3.4	35.5 (29.8–42.4)	34.8 ± 3.4	33.3 (29.3–41.6)	0.537
TWB, %	44.5 ± 3.5	45.8 (38.3–49.3)	43.6 ± 2.7	43.6 (37.5–47.9)	0.328
ECW, L	16.7 ± 1.4	16.8 (14.3–19.7)	16.7 ± 1.6	16.8 (14.2–20.0)	0.862
ECW, % of TBW	47.3 ± 2.6	46.8 (43.7–53.8)	48.0 ± 2.5	48.1 (43.2–52.9)	0.438
ICW, L	18.7 ± 2.4	18.9 (14.2–22.9)	18.1 ± 2.2	18.0 (15.2–21.7)	0.419
ICW, % of TBW	52.7 ± 2.7	53.3 (46.3–56.3)	52.0 ± 2.4	51.9 (47.1–56.5)	0.428

* Non-normal distribution (verified using Shapiro–Wilk test; *p* ≤ 0.05); ** Student’s *t*-test or U Mann–Whitney test (depending on data distribution); Na/K ratio—sodium/potassium ratio; FM—fat mass; FFM—fat-free mass; BCM—body cell mass; BCMI—body cell mass index; MM—muscle mass; TBW—total body water; ECW—extracellular water; ICW—intracellular water.

**Table 7:** The energy value and macronutrient intake in the diets of the participants from the intervention and the control group at baseline.

Diet Characteristics	Intervention Group (*n* = 19)		Control Group (*n* = 19)		p **
	Mean ± SD	Median (Min–Max)	Mean ± SD	Median (Min–Max)	
Energy, kcal	1894 ± 459	1834 (891–2718)	1791 ± 449	1827 (962–2397)	0.488
Total protein, g	76.1 ± 16.7	74.6 (48.9–111.5)	75.0 ± 20.4	71.5 (42.5–113.6)	0.858
Animal protein, g	49.9 ± 16.1	42.3 (28.5–80.1) *	48.1 ± 16.2	47.8 (23.1–81.4)	0.827
Plant protein, g	24.9 ± 6.6	25.4 (11.7–37.4)	25.6 ± 11.0	24.0 (10.0–52.8)	0.830
Animal protein/plant protein ratio	2.2 ± 0.9	2.2 (1.0–3.7)	2.2 ± 1.2	2.0 (0.5–6.2) *	0.885
Fat, g	78.5 ± 26.9	76.7 (32.3–136.2)	72.1 ± 17.7	82.3 (43.5–94.3) *	0.452
SFA, g	29.3 ± 9.8	27.3 (10.5–48.7)	26.7 ± 7.3	25.2 (13.4–40.2)	0.364
MUFA, g	30.2 ± 12.1	27.9 (13.8–61.6)	29.0 ± 8.4	29.8 (15.0–39.5)	0.715
PUFA, g	12.9 ± 7.1	11.6 (2.8–32.8) *	12.8 ± 5.2	11.2 (4.8–23.0)	0.708
Omega-6 fatty acids, g	10.5 ± 6.2	8.7 (2.4–28.2) *	10.2 ± 4.1	9.4 (3.6–20.0)	0.644
Omega-3 fatty acids, g	2.4 ± 1.2	2.3 (0.4–4.7)	2.6 ± 1.5	2.3 (0.7–6.0)	0.743
Omega-6/omega-3 ratio	4.5 ± 1.7	4.2 (2.6–8.2)	4.7 ± 2.6	4.2 (2.0–12.9) *	0.954
LA (C18:2n6), g	10.3 ± 6.2	8.6 (2.3–28.0) *	10.1 ± 4.1	9.4 (3.5–19.8)	0.603
ALA (C18:3n3), g	1.9 ± 0.8	2.0 (0.4–3.2)	2.2 ± 1.1	2.2 (0.5–4.0)	0.414
EPA (C20:5n3), mg	128 ± 137	83 (3–433) *	108 ± 234	10 (0–830) *	0.754
DPA (C22:5n3), mg	52 ± 50	33 (3–180) *	35 ± 60	10 (0–250) *	0.350
DHA(C22:6n3), mg	323 ± 345	233 (23–1367) *	254 ± 464	57 (10–1800) *	0.610
Cholesterol, g	354.5 ± 136.4	324.1 (178.0–698.6)	285.8 ± 120.0	284.7 (107.6–557.6)	0.108
Carbohydrates, g	217.6 ± 51.0	217.2 (92.4–305.2)	215.1 ± 64.3	227.8 (99.1–301.0)	0.895
Glucose, g	6.2 ± 3.0	6.0 (1.3–13.2)	7.6 ± 5.3	7.1 (1.3–21.0) *	0.644
Fructose, g	7.2 ± 3.8	7.3 (1.2–17.3)	8.7 ± 6.7	6.3 (1.3–26.1) *	0.885
Sucrose, g	34.1 ± 18.4	30.4 (5.3–72.7)	36.6 ± 23.9	35.2 (5.8–92.7) *	0.908
Lactose, g	7.4 ± 6.2	6.0 (1.3–23.5) *	6.6 ± 5.1	5.6 (1.0–20.5) *	0.795
Fiber, g	16.9 ± 5.1	16.4 (10.2–27.1)	18.6 ± 8.5	17.3 (6.6–38.0)	0.665
Energy from protein, %	16.5 ± 3.1	16.6 (10.9–22.0)	17.0 ± 3.2	15.8 (12.3–24.8)	0.663
Energy from fats, %	36.9 ± 6.9	37.8 (24.3–47.8)	36.5 ± 4.6	36.1 (29.0–44.7)	0.849
Energy from carbohydrates, %	46.6 ± 7.5	45.3 (34.4–64.3)	46.5 ± 6.2	46.6 (36.1–55.7)	0.971

* Non-normal distribution (verified using Shapiro–Wilk test; *p* ≤ 0.05); ** Student’s *t*-test or U Mann–Whitney test (depending on data distribution); SFA—saturated fatty acids; MUFA—monounsaturated fatty acids; PUFA—polyunsaturated fatty acids; Omega-6 fatty acids—the sum of omega-6 fatty acids; Omega-3 fatty acids—the sum of omega-3 fatty acids; LA—linoleic acid; ALA—alpha-linolenic acid; EPA—eicosapentaenoic acid; DPA—docosapentaenoic acid; DHA—docosahexaenoic acid.

**Table 8:** Lipid profile and the calculated atherogenic indices of participants from the intervention and the control group at baseline, during the intervention (after 4 weeks of intervention, in week 5), and after the intervention (in week 9).

Measurement Time	Lipid Profile Components	Intervention Group (*n* = 19)		Control Group (*n* = 19)		p **
		Mean ± SD	Median (Min–Max)	Mean ± SD	Median (Min–Max)	
Baseline (w0)	TC, mg/dL	185.4 ± 42.8	171.9 (130.3–318.5) *	180.2 ± 29.7	174.7 (142.1–236.3)	0.885
	HDL, mg/dL	59.0 ± 14.2	58.1 (36.9–87.4)	57.4 ± 10.8	55.9 (38.9–84.0)	0.701
	Non-HDL, mg/dL	126.5 ± 43.1	124.2 (71.1–251.7) *	122.9 ± 31.4	112.9 (86.7–189.5) *	0.885
	LDL, mg/dL	105.9 ± 38.4	101.4 (57.7–220.0) *	98.7 ± 25.7	89.3 (73.0–159.2) *	0.644
	TAG, mg/dL	101.3 ± 35.3	89.9 (46.0–185.3)	120.6 ± 62.3	99.4 (53.9–267.0) *	0.549
	AIP	−0.14 ± 0.22	−0.15 (−0.48–0.34)	−0.08 ± 0.22	−0.13 (−0.35–0.38)	0.385
	AC	2.30 ± 1.04	2.15 (0.89–5.17)	2.25 ± 0.84	1.84 (1.30–4.05) *	1.000
	Cholindex, mg/dL	46.9 ± 42.5	48.1 (−21.9–153.2)	41.4 ± 31.1	29.8 (6.3–103.8) *	0.544
	CRI-1	3.30 ± 1.04	3.15 (1.89–6.17)	3.25 ± 0.84	2.84 (2.30–5.05) *	1.000
	CRI-2	1.92 ± 0.86	1.84 (0.73–4.16)	1.81 ± 0.69	1.50 (1.09–3.05) *	0.686
	TAG/HDL	0.82 ± 0.45	0.70 (0.33–2.19) *	0.96 ± 0.56	0.73 (0.45–2.39) *	0.470
During the intervention (w5)	TC, mg/dL	186.2 ± 36.1	177.5 (136.4–272.9)	183.9 ± 35.6	184.4 (116.8–245.4)	0.839
	HDL, mg/dL	61.4 ± 15.5	58.9 (36.6–88.8)	59.2 ± 14.8	60.6 (32.4–86.6)	0.657
	Non-HDL, mg/dL	124.8 ± 35.7	113.3 (69.9–204.1)	124.7 ± 34.9	108.0 (76.8–183.1)	0.988
	LDL, mg/dL	103.1 ± 33.0	94.5 (59.3–170.8)	101.4 ± 31.0	89.6 (48.1–166.5)	0.864
	TAG, mg/dL	108.4 ± 41.3	107.6 (42.8–194.6)	116.5 ± 54.1	98.9 (55.1–265.8) *	0.954
	AIP	−0.13 ± 0.23	−0.05 (−0.56–0.31)	−0.08 ± 0.23	−0.12 (−0.41–0.55)	0.539
	AC	2.20 ± 0.96	1.94 (0.85–4.42) *	2.29 ± 1.08	1.92 (1.22–5.55) *	1.000
	Cholindex, mg/dL	41.7 ± 38.5	32.4 (−22.5–125.6)	42.2 ± 35.4	27.2 (−4.7–104.2)	0.973
	CRI-1	3.20 ± 0.96	2.94 (1.85–5.42) *	3.29 ± 1.08	2.92 (2.22–6.55) *	1.000
	CRI-2	1.81 ± 0.84	1.64 (0.73–3.86) *	1.85 ± 0.84	1.44 (0.95–3.91) *	0.977
	TAG/HDL	0.84 ± 0.42	0.89 (0.27–2.05)	0.97 ± 0.73	0.75 (0.39–3.58) *	0.908
After the intervention (w9)	TC, mg/dL	181.9 ± 40.0	176.6 (125.8–287.5)	188.1 ± 33.4	185.7 (142.3–258.1)	0.610
	HDL, mg/dL	57.7 ± 14.0	56.5 (34.8–83.2)	57.7 ± 10.0	58.0 (40.8–78.4)	0.997
	Non-HDL, mg/dL	124.3 ± 40.8	120.2 (71.9–214.6)	130.4 ± 32.0	132.4 (82.1–199.7)	0.609
	LDL, mg/dL	97.7 ± 37.7	99.1 (21.8–191.3)	108.2 ± 28.1	105.5 (67.2–171.3)	0.337
	TAG, mg/dL	132.9 ± 73.8	122.1 (58.0–365.4) *	111.0 ± 41.8	103.1 (49.5–172.8)	0.525
	AIP	−0.03 ± 0.28	−0.01 (−0.41–0.66)	−0.10 ± 0.20	−0.09 (−0.48–0.26)	0.395
	AC	2.34 ± 1.14	1.99 (0.91–5.66) *	2.33 ± 0.71	2.10 (1.26–3.88)	0.644
	Cholindex, mg/dL	40.0 ± 41.1	52.3 (−32.1–118.4)	50.5 ± 29.8	49.5 (5.7–112.9)	0.374
	CRI-1	3.34 ± 1.14	2.99 (1.91–6.66) *	3.33 ± 0.71	3.10 (2.26–4.88)	0.644
	CRI-2	1.82 ± 0.85	1.75 (0.40–3.56)	1.93 ± 0.60	1.91 (1.07–3.04)	0.638
	TAG/HDL	1.15 ± 0.96	0.98 (0.39–4.58) *	0.87 ± 0.38	0.82 (0.33–1.84)	0.506

* Non-normal distribution (verified using Shapiro–Wilk test; *p* ≤ 0.05); ** Student’s *t*-test or U Mann–Whitney test (depending on data distribution); w0—baseline; w5—after 4 weeks of intervention, in week 5; w9—after 8 weeks of intervention, in week 9; TC—total cholesterol; HDL—high-density lipoprotein; LDL—low-density lipoprotein; TAG—triglycerides; AIP—Atherogenic Index of Plasma; AC—Atherogenic Coefficient; Cholindex—Cholesterol Index; CRI-1—Castelli Risk Index 1; CRI-2—Castelli Risk Index 2.

**Table 9:** Lipid profile changes from baseline to the middle of the intervention (from w0 to w5), from the middle to after the intervention (from w5 to w9), and from baseline to after the intervention (from w0 to w9).

Period of Change	Lipid Profile Components	Intervention Group (*n* = 19)		Control Group (*n* = 19)		p **
		Mean ± SD	Median (Min–Max)	Mean ± SD	Median (Min–Max)	
Change from w0 to w5	TC, mg/dL	0.8 ± 18.4	1.0 (−45.6–31.3)	3.6 ± 18.8	6.0 (−25.3–38.8)	0.645
	HDL, mg/dL	2.5 ± 6.1	1.3 (−5.8–18.1)	1.8 ± 6.9	4.6 (−14.4–12.3)	0.770
	Non-HDL, mg/dL	−1.6 ± 17.3	−1.2 (−47.6–24.2)	1.8 ± 13.2	1.3 (−16.6–30.8)	0.498
	LDL, mg/dL	−2.7 ± 16.7	−3.5 (−49.2–22.3)	2.6 ± 17.5	0.0 (−33.3–43.2)	0.342
	TAG, mg/dL	7.2 ± 40.8	−1.1 (−75.9–106.2)	−4.1 ± 37.9	−8.2 (−109.5–89.8) *	0.370
Change from w5 to w9	TC, mg/dL	−4.3 ± 19.4	−0.3 (−46.4–32.0)	4.2 ± 28.1	5.1 (−57.3–41.0)	0.283
	HDL, mg/dL	−3.7 ± 7.9	−2.5 (−24.0–10.0)	−1.5 ± 10.8	−1.5 (−19.7–21.3)	0.473
	Non-HDL, mg/dL	−0.6 ± 18.3	4.9 (−26.3–42.6)	5.8 ± 23.6	4.5 (−41.2–46.1)	0.364
	LDL, mg/dL	−5.5 ± 21.4	−5.0 (−45.8–41.6)	6.8 ± 21.7	0.4 (−44.4–40.2)	0.087
	TAG, mg/dL	24.4 ± 67.4	22.0 (−61.1–256.0)	−5.5 ± 46.2	4.2 (−94.1–58.6)	0.234
Change from w0 to w9	TC, mg/dL	−3.5 ± 20.8	−3.2 (−40.0–46.4) *	7.9 ± 22.9	12.8 (−37.3–49.4)	0.199
	HDL, mg/dL	−1.3 ± 8.7	−2.1 (−22.6–18.0)	0.3 ± 9.4	−1.3 (−12.7–25.9)	0.590
	Non-HDL, mg/dL	−2.2 ± 17.4	−1.4 (−37.1–38.9)	7.5 ± 19.3	7.4 (−31.3–41.3)	0.111
	LDL, mg/dL	−8.2 ± 20.7	−7.9 (−48.0–38.2)	9.5 ± 20.0	8.0 (−14.4–60.1)	0.011
	TAG, mg/dL	31.6 ± 57.2	17.0 (−42.0–180.1) *	−9.6 ± 49.3	−7.3 (−105.3–68.4)	0.053

* Non-normal distribution (verified using Shapiro–Wilk test; *p* ≤ 0.05); ** Student’s *t*-test or U Mann–Whitney test (depending on data distribution); w0—baseline; w5—after 4 weeks of intervention, in week 5; w9—after 8 weeks of intervention, in week 9; TC—total cholesterol; HDL—high-density lipoprotein; LDL—low-density lipoprotein; TAG—triglycerides.

**Table 10:** Lipid profile changes from baseline to the middle of the intervention (from w0 to w5), from the middle to after the intervention (from w5 to w9), and from baseline to after the intervention (from w0 to w9) across overweight participants (BMI 25.0–29.9 kg/m^2^).

Period of Change	Lipid Profile Components	Intervention Group (*n* = 14)		Control Group (*n* = 15)		p **
		Mean ± SD	Median (Min–Max)	Mean ± SD	Median (Min–Max)	
Change from w0 to w5	TC, mg/dL	3.7 ± 15.4	2.7 (−16.4–31.3)	6.3 ± 18.5	6.0 (−25.3–38.8)	0.693
	HDL, mg/dL	1.9 ± 6.8	1.0 (−5.8–18.1) *	3.0 ± 5.8	4.7 (−10.0–12.3)	0.371
	Non-HDL, mg/dL	1.9 ± 14.2	−1.2 (−13.9–24.2)	3.3 ± 14.0	1.3 (−16.6–30.8)	0.782
	LDL, mg/dL	0.0 ± 13.3	−0.3 (−20.8–22.3)	3.7 ± 19.2	0.0 (−33.3–43.2)	0.554
	TAG, mg/dL	9.3 ± 30.6	−1.0 (−49.7–64.1)	−1.9 ± 41.9	−7.7 (−109.5–89.8)	0.424
Change from w5 to w9	TC, mg/dL	−5.0 ± 19.2	−0.9 (−46.4–32.0)	5.8 ± 30.9	6.2 (−57.3–41.0)	0.270
	HDL, mg/dL	−3.3 ± 8.5	−2.5 (−24.0–10.0)	−2.5 ± 9.0	−3.0 (−16.1–15.0)	0.805
	Non-HDL, mg/dL	−1.7 ± 19.0	0.1 (−25.0–42.6)	8.3 ± 25.5	6.6 (−41.2–46.1)	0.245
	LDL, mg/dL	−3.6 ± 18.5	−3.6 (−39.5–41.6)	8.9 ± 24.1	12.1 (−44.4–40.2)	0.131
	TAG, mg/dL	9.4 ± 37.4	6.0 (−61.1–85.5)	−3.1 ± 41.5	4.2 (−76.9–58.6)	0.404
Change from w0 to w9	TC, mg/dL	−1.3 ± 22.5	−3.6 (−40.0–46.4) *	12.1 ± 20.9	15.2 (−20.2–49.4)	0.169
	HDL, mg/dL	−1.4 ± 10.0	−1.6 (−22.6–18.0)	0.5 ± 6.9	0.0 (−9.2–9.3)	0.556
	Non-HDL, mg/dL	0.1 ± 17.3	−2.3 (−24.0–38.9)	11.6 ± 18.3	10.3 (−16.1–41.3)	0.095
	LDL, mg/dL	−3.6 ± 19.1	−7.1 (−34.3–38.2)	12.6 ± 20.7	11.8 (−12.4–60.1)	0.038
	TAG, mg/dL	18.6 ± 29.9	14.2 (−14.4–84.7)	−5.0 ± 47.5	−7.3 (−105.3–68.4)	0.124

* Non-normal distribution (verified using Shapiro–Wilk test; *p* ≤ 0.05); ** Student’s *t*-test or U Mann–Whitney test (depending on data distribution); w0—baseline; w5—after 4 weeks of intervention, in week 5; w9—after 8 weeks of intervention, in week 9; TC—total cholesterol; HDL—high-density lipoprotein; LDL—low-density lipoprotein; TAG—triglycerides.

**Table 11:** Lipid profile changes from baseline to the middle of the intervention (from w0 to w5), from the middle to after the intervention (from w5 to w9), and from baseline to after the intervention (from w0 to w9) across participants with a WHtR > 0.5.

Period of Change	Lipid Profile Components	Intervention Group (*n* = 11)		Control Group (*n* = 11)		p **
		Mean ± SD	Median (Min–Max)	Mean ± SD	Median (Min–Max)	
Change from w0 to w5	TC, mg/dL	4.1 ± 21.2	6.4 (−45.6–31.3)	4.6 ± 21.2	8.7 (−24.2–38.8)	0.956
	HDL, mg/dL	2.9 ± 5.0	2.0 (−5.8–12.8)	1.3 ± 7.1	4.6 (−14.4–8.0) *	1.000
	Non-HDL, mg/dL	1.2 ± 20.1	2.4 (−47.6–23.6) *	3.2 ± 15.4	3.6 (−16.6–30.8)	0.949
	LDL, mg/dL	−0.2 ± 19.9	2.9 (−49.2–22.3)	5.7 ± 18.2	5.2 (−13.4–43.2)	0.479
	TAG, mg/dL	9.9 ± 48.5	−0.8 (−75.9–106.2)	−12.1 ± 37.3	−8.4 (−109.5–35.8) *	0.401
Change from w5 to w9	TC, mg/dL	−8.9 ± 20.8	−3.4 (−46.4–16.2)	−0.8 ± 31.2	5.1 (−57.3–41.0)	0.480
	HDL, mg/dL	−4.3 ± 8.6	−3.3 (−24.0–6.8)	−3.5 ± 11.9	−6.0 (−19.7–21.3)	0.846
	Non-HDL, mg/dL	−4.6 ± 17.2	2.2 (−26.3–21.5)	2.7 ± 28.1	4.5 (−41.2–46.1)	0.473
	LDL, mg/dL	−12.6 ± 22.2	−11.6 (−45.8–20.5)	2.0 ± 22.5	−2.3 (−44.4–35.8)	0.141
	TAG, mg/dL	40.2 ± 81.7	30.1 (−49.8–256.0) *	3.5 ± 52.6	16.0 (−94.1–58.6)	0.519
Change from w0 to w9	TC, mg/dL	−4.8 ± 20.8	−2.1 (−40.0–38.4)	3.8 ± 23.5	15.2 (−37.3–30.6)	0.372
	HDL, mg/dL	−1.5 ± 8.2	−0.3 (−22.6–8.3) *	−2.1 ± 10.8	−6.0 (−12.7–25.9) *	0.217
	Non-HDL, mg/dL	−3.4 ± 17.0	−1.4 (−37.1–30.1)	5.9 ± 21.1	8.1 (−31.3–39.2)	0.269
	LDL, mg/dL	−12.8 ± 21.8	−10.1 (−48.0–27.8)	7.7 ± 18.1	12.1 (−14.4–36.3)	0.027
	TAG, mg/dL	50.0 ± 68.3	32.9 (−42.0–180.1)	−8.6 ± 56.6	7.6 (−105.3–68.4)	0.040

* Non-normal distribution (verified using Shapiro–Wilk test; *p* ≤ 0.05); ** Student’s *t*-test or U Mann–Whitney test (depending on data distribution); w0—baseline; w5—after 4 weeks of intervention, in week 5; w9—after 8 weeks of intervention, in week 9; TC—total cholesterol; HDL—high-density lipoprotein; LDL—low-density lipoprotein; TAG—triglycerides.

**Table 12:** Number of participants with lipid profile components considered as normal vs. elevated/low at baseline, during the intervention (after 4 weeks of intervention, in week 5), and after the intervention (in week 9).

		Intervention Group (*n* = 19)			Control Group (*n* = 19)			*p* *
Measurement Time	Lipid Profile Components	Normal Concentration *n* (%)	Elevated Concentration *n* (%)	Low Concentration *n* (%)	Normal Concentration *n* (%)	Elevated Concentration *n* (%)	Low Concentration *n* (%)	
Baseline (w0)	TC	13 (68%)	6 (32%)	–	13 (68%)	6 (32%)	–	1.000
	HDL	15 (79%)	–	4 (21%)	17 (89%)	–	2 (11%)	0.656
	Non-HDL	15 (79%)	4 (21%)	–	14 (74%)	5 (26%)	–	1.000
	LDL	13 (68%)	6 (32%)	–	14 (74%)	5 (26%)	–	1.000
	TAG	17 (89%)	2 (11%)	–	15 (79%)	4 (21%)	–	0.656
During the intervention (w5)	TC	10 (53%)	9 (47%)	–	11 (58%)	8 (42%)	–	1.000
	HDL	15 (79%)	–	4 (21%)	15 (79%)	–	4 (21%)	1.000
	Non-HDL	13 (68%)	6 (32%)	–	14 (74%)	5 (26%)	–	1.000
	LDL	12 (63%)	7 (37%)	–	10 (53%)	9 (47%)	–	0.742
	TAG	16 (84%)	3 (16%)	–	17 (89%)	2 (11%)	–	1.000
After the intervention (w9)	TC	13 (68%)	6 (32%)	–	12 (63%)	7 (37%)	–	1.000
	HDL	15 (79%)	–	4 (21%)	18 (95%)	–	1 (5%)	0.337
	Non-HDL	14 (74%)	5 (26%)	–	14 (74%)	5 (26%)	–	1.000
	LDL	13 (68%)	6 (32%)	–	12 (63%)	7 (37%)	–	1.000
	TAG	15 (79%)	4 (21%)	–	15 (79%)	4 (21%)	–	1.000

* Chi^2^ test with Yates’ correction; w0—baseline; w5—after 4 weeks of intervention, in week 5; w9—after 8 weeks of intervention, in week 9; TC—total cholesterol; HDL—high-density lipoprotein; LDL—low-density lipoprotein; TAG—triglycerides; normal concentration of TC: <190.0 mg/dL; elevated concentration of TC: >190 mg/dL; normal concentration of HDL: >45.0 mg/dL; low concentration of HDL: <45 mg/dL; normal concentration of non-HDL: <145.0 mg/dL; elevated concentration of non-HDL: >145.0 mg/dL; normal concentration of LDL: <115.0 mg/dL; elevated concentration of LDL: >115.0 mg/dL; normal concentration of TAG: <150.0 mg/dL; elevated concentration of TAG: >150 mg/dL.

**Table 13:** Number of participants for whom a positive change was observed considering a decrease (of TC, non-HDL, LDL, TAG) or an increase (of HDL) in the concentration of the specific lipid profile components from baseline to after 8 weeks of the intervention (in week 9).

	Intervention Group (*n* = 19)		Control Group (*n* = 19)		*p* *
Lipid Profile Components	Positive Change *n* (%)	Negative Change *n* (%)	Positive Change *n* (%)	Negative Change *n* (%)	
TC decrease from w0 to w9	14 (74%)	5 (26%)	8 (42%)	11 (58%)	0.100
HDL increase from w0 to w9	8 (42%)	11 (58%)	8 (42%)	11 (58%)	1.000
Non-HDL decrease from w0 to w9	10 (53%)	9 (47%)	8 (42%)	11 (58%)	0.745
LDL decrease from w0 to w9	12 (63%)	7 (37%)	7 (37%)	12 (63%)	0.194
TAG decrease from w0 to w9	6 (32%)	13 (68%)	11 (58%)	8 (42%)	0.192

* Chi^2^ test with Yates’ correction; w0—baseline; w5—after 4 weeks of intervention, in week 5; w9—after 8 weeks of intervention, in week 9; TC—total cholesterol; HDL—high-density lipoprotein; LDL—low-density lipoprotein; TAG—triglycerides; positive change—defined as a decrease in a lipid profile component concentration from w0 to w9 (change lower than 0 mg/dL) as for: TC, non-HDL, LDL and TAG, or an increase in the concentration of HDL from w0 to w9 (change greater than 0 mg/dL).

**Table 14:** The changes in the analyzed atherogenic indices during the study (from baseline to after intervention, w0 to w9).

Atherogenic Index	Intervention Group (*n* = 19)		Control Group (*n* = 19)		p **
	Mean ± SD	Median (Min–Max)	Mean ± SD	Median (Min–Max)	
AIP	0.10 ± 0.18	0.08 (−0.21–0.48)	−0.03 ± 0.22	−0.07 (−0.56–0.36)	0.054
AC	0.04 ± 0.41	0.13 (−1.00–0.54) *	0.08 ± 0.50	0.16 (−1.41–0.96) *	0.977
Cholindex, mg/dL	−6.9 ± 19.9	−4.4 (−43.8–30.7)	9.1 ± 18.4	0.8 (−12.3–52.0) *	0.040
CRI−1	0.04 ± 0.41	0.13 (−1.00–0.54) *	0.08 ± 0.50	0.16 (−1.41–0.96) *	0.977
CRI−2	−0.10 ± 0.41	−0.02 (−0.87–0.48)	0.12 ± 0.40	0.06 (−0.93–0.75)	0.099
TAG/HDL	0.32 ± 0.63	0.16 (−0.34–2.39) *	−0.09 ± 0.48	−0.09 (−1.06–0.69)	0.057

* Non-normal distribution (verified using Shapiro–Wilk test; *p* ≤ 0.05); ** Student’s *t*-test or U Mann–Whitney test (depending on data distribution); AIP—atherogenic index of plasma; AC—atherogenic coefficient; Cholindex—cholesterol index; CRI-1—Castelli risk index 1; CRI-2—Castelli risk index 2; TAG—triglycerides; HDL—high-density lipoprotein.

## Data Availability

Data are available on request from the corresponding author. The data are not publicly available due to ethical reasons.

## Supplementary Materials

The following supporting information can be downloaded at: https://www.mdpi.com/article/10.3390/nu16234051/s1, Table S1: Lipid profile changes from baseline to the middle of the intervention (from w0 to w5), from the middle to after the intervention (from w5 to w9), and from baseline to after the intervention (from w0 to w9) across obese participants (BMI ≥ 30.0 kg/m^2^); Table S2: Lipid profile changes from baseline to the middle of the intervention (from w0 to w5), from the middle to after the intervention (from w5 to w9), and from baseline to after the intervention (from w0 to w9) across participants with a WHtR ≤ 0.5.
