# Supplementary material for: Effects of Increasing Farmed Salmon Intake to the Recommended Fish-Intake Amounts on Lipid Profile in Young Women: An 8-Week Intervention Study
Source: Nutrients. 2024 Nov 26;16(23):4051. doi: 10.3390/nu16234051 (PMC11643451; doi:10.3390/nu16234051)
Supplement: Supplementary file 1 [file nutrients-16-04051-s001.zip › nutrients-3318907-supplementary.pdf]

nutrients

MDPI

Supplementary Materials

Effects of Increasing Farmed Salmon Intake to the Recommended Fish-Intake Amounts on Lipid Profile in Young Women: An 8-Week Intervention Study

Zofia Utri-Khodadady, Dominika Głąbska

Table S1. Lipid profile changes from baseline to the middle of the intervention (from w0 to w5), from the middle to after the intervention (from w5 to w9), and from baseline to after the intervention (from w0 to w9) across obese participants (BMI≥30.0 kg/m²).

Period of change	Lipid profile components	Smoked salmon group (*n*=5)		Control group (*n*=4)		*p***
		Mean ± SD	Median (Min – Max)	Mean ± SD	Median (Min – Max)	
Change from w0 to w5	TC, mg/dL	-7.3 ± 25.2	1.0 (-45.6; 18.5)	-6.3 ± 18.5	-5.9 (-24.2; 10.6)	0.949
	HDL, mg/dL	4.1 ± 3.1	2.2 (1.3; 7.8)	-2.4 ± 10.0	-1.1 (-14.4; 7.0)	0.461
	Non-HDL, mg/dL	-11.4 ± 23.0	-1.2 (-47.6; 10.7)	-4.0 ± 9.2	-3.1 (-13.7; 4.1)	0.565
	LDL, mg/dL	-10.4 ± 24.2	-9.8 (-49.2; 15.9)	-1.5 ± 9.9	-1.4 (-11.7; 8.6)	0.514
	TAG, mg/dL	1.3 ± 66.3	-14.1 (-75.9; 106.2)	-12.5 ± 16.9	-15.4 (-28.5; 9.4)	0.702
Change from w5 to w9	TC, mg/dL	-2.3 ± 22.0	-0.3 (-38.3; 16.2)	-1.6 ± 15.2	-4.0 (-15.2; 16.7)	0.960
	HDL, mg/dL	-5.0 ± 6.2	-5.3 (-12.0; 4.1)	2.1 ± 17.3	3.5 (-19.7; 21.3)	0.417
	Non-HDL, mg/dL	2.7 ± 17.8	5.4 (-26.3; 21.5)	-3.8 ± 12.8	-0.1 (-21.5; 6.6)	0.565
	LDL, mg/dL	-10.7 ± 30.2	-6.3 (-45.8; 20.5)	-0.9 ± 4.0	-2.5 (-3.6; 5.0)*	0.730
	TAG, mg/dL	66.6 ± 113.6	42.6 (-49.8; 256.0)	-14.3 ± 68.2	-3.9 (-94.1; 44.6)	0.253
Change from w0 to w9	TC, mg/dL	-9.6 ± 15.1	-2.1 (-31.0; 4.0)	-8.0 ± 26.1	-10.0 (-37.3; 25.4)	0.906
	HDL, mg/dL	-0.9 ± 4.2	-2.1 (-4.2; 6.1)	-0.3 ± 17.7	-7.1 (-12.7; 25.9)	0.286
	Non-HDL, mg/dL	-8.7 ± 17.9	1.0 (-37.1; 6.1)	-7.7 ± 16.9	-3.8 (-31.3; 8.1)	0.932
	LDL, mg/dL	-21.1 ± 21.6	-28.7 (-48.0; 6.1)	-2.4 ± 12.6	-4.3 (-14.4; 13.6)	0.172
	TAG, mg/dL	67.8 ± 98.0	28.5 (-42.0; 180.1)	-26.8 ± 59.4	-27.3 (-84.7; 32.2)	0.136

*non-normal distribution (verified using Shapiro–Wilk test; $p \leq 0.05$); **t-Student test or U Mann-Whitney test (depending on data distribution); w0—baseline; w5—after 4 weeks of intervention, in week 5; w9—after 8 weeks of intervention, in week 9; TC – total cholesterol; HDL – high-density lipoprotein; LDL – low-density lipoprotein; TAG – triglycerides.

Table S2. Lipid profile changes from baseline to the middle of the intervention (from w0 to w5), from the middle to after the intervention (from w5 to w9), and from baseline to after the intervention (from w0 to w9) across participants with a WHtR≤0.5.

		**Smoked salmon group (*n*=9)**		**Control group (*n*=9)**		p**
		Mean ± SD	Median (Min – Max)	Mean ± SD	Median (Min – Max)	
Change from w0 to w5	TC, mg/dl	-3.6 ± 13.7	-8.5 (-16.4; 19.8)	2.3 ± 16.2	3.8 (-25.3; 22.0)	0.441
	HDL, mg/dl	1.9 ± 7.7	-0.8 (-4.4; 18.1)*	2.5 ± 7.1	3.7 (-10.0; 12.3)	0.721
	Non-HDL, mg/dl	-5.5 ± 12.6	-9.7 (-13.9; 24.2)*	-0.2 ± 10.3	-0.3 (-15.3; 16.5)	0.195
	LDL, mg/dl	-6.2 ± 11.2	-10.0 (-20.8; 14.2)	-1.6 ± 16.8	-0.2 (-33.3; 20.0)	0.528
	TAG, mg/dl	3.4 ± 29.9	-1.2 (-49.7; 49.9)	6.9 ± 38.3	-2.2 (-36.3; 89.8)	0.842
Change from w5 to w9	TC, mg/dl	2.0 ± 16.3	4.5 (-19.9; 32.0)	11.1 ± 23.4	10.9 (-16.5; 37.7)*	0.645
	HDL, mg/dl	-2.9 ± 7.3	-2.5 (-11.5; 10.0)	1.2 ± 9.1	-0.5 (-10.0; 15.0)	0.339
	Non-HDL, mg/dl	4.9 ± 19.5	7.2 (-20.9; 42.6)	9.9 ± 16.7	11.1 (-8.4; 32.7)	0.590
	LDL, mg/dl	4.4 ± 17.0	0.8 (-11.8; 41.6)	13.5 ± 20.0	9.2 (-9.0; 40.2)	0.342
	TAG, mg/dl	2.8 ± 35.1	3.0 (-61.1; 46.4)	-17.8 ± 35.3	-9.0 (-76.9; 16.0)	0.261
Change from w0 to w9	TC, mg/dl	-1.6 ± 22.0	-3.6 (-32.7; 46.4)*	13.4 ± 22.2	12.6 (-11.2; 49.4)	0.328
	HDL, mg/dl	-1.0 ± 10.0	-4.2 (-14.0; 18.0)	3.7 ± 6.3	6.6 (-7.4; 9.3)	0.280
	Non-HDL, mg/dl	-0.6 ± 19.0	-0.8 (-24.0; 38.9)	9.7 ± 17.6	5.5 (-7.1; 41.3)	0.278
	LDL, mg/dl	-1.8 ± 18.7	-7.1 (-21.1; 38.2)	11.9 ± 23.4	4.9 (-12.4; 60.1)	0.214
	TAG, mg/dl	6.2 ± 22.0	-1.6 (-14.4; 50.9)	-10.9 ± 40.8	-10.8 (-94.2; 43.2)	0.314

*non-normal distribution (verified using Shapiro–Wilk test; $p \leq 0.05$); **t-student test or U Mann-Whitney test; w0—baseline; w5—after 4 weeks of intervention, in week 5; w9—after 8 weeks of intervention, in week 9; TC – total cholesterol; HDL – high-density lipoprotein; LDL – low-density lipoprotein; TAG – triglycerides.
